# Has Tanzania Embraced the Green Leaf? Results from Outlet and Household Surveys before and after Implementation of the Affordable Medicines Facility -Malaria

**DOI:** 10.1371/journal.pone.0095607

**Published:** 2014-05-09

**Authors:** Rebecca Thomson, Charles Festo, Boniface Johanes, Admirabilis Kalolella, Katia Bruxvoort, Happy Nchimbi, Sarah Tougher, Matthew Cairns, Mark Taylor, Immo Kleinschmidt, Yazoume Ye, Andrea Mann, Ruilin Ren, Barbara Willey, Fred Arnold, Kara Hanson, S. Patrick Kachur, Catherine Goodman

**Affiliations:** 1 London School of Hygiene & Tropical Medicine, London, United Kingdom; 2 Ifakara Health Institute, Dar es Salaam, Tanzania; 3 Department of Public Health, Trnava University, Trnava, Slovakia; 4 International Health Division, ICF International, Calverton, Maryland, United States of America; 5 Malaria Branch, Centers for Disease Control & Prevention, Atlanta, Georgia, United States of America; Johns Hopkins University, United States of America

## Abstract

**Background:**

The Affordable Medicines Facility - malaria (AMFm) is primarily an artemisinin combination therapy (ACT) subsidy, aimed at increasing availability, affordability, market share and use of quality-assured ACTs (QAACTs). Mainland Tanzania was one of eight national scale programmes where AMFm was introduced in 2010. Here we present findings from outlet and household surveys before and after AMFm implementation to evaluate its impact from both the supply and demand side.

**Methods:**

Outlet surveys were conducted in 49 randomly selected wards throughout mainland Tanzania in 2010 and 2011, and data on outlet characteristics and stocking patterns were collected from outlets stocking antimalarials. Household surveys were conducted in 240 randomly selected enumeration areas in three regions in 2010 and 2012. Questions about treatment seeking for fever and drugs obtained were asked of individuals reporting fever in the previous two weeks.

**Results:**

The availability of QAACTs increased from 25.5% to 69.5% among all outlet types, with the greatest increase among pharmacies and drug stores, together termed specialised drug sellers (SDSs), where the median QAACT price fell from $5.63 to $0.94. The market share of QAACTs increased from 26.2% to 42.2%, again with the greatest increase in SDSs. Household survey results showed a shift in treatment seeking away from the public sector towards SDSs. Overall, there was no change in the proportion of people with fever obtaining an antimalarial or ACT from baseline to endline. However, when broken down by treatment source, ACT use increased significantly among clients visiting SDSs.

**Discussion:**

Unchanged ACT use overall, despite increases in QAACT availability, affordability and market share in the private sector, reflected a shift in treatment seeking towards private providers. The reasons for this shift are unclear, but likely reflect both persistent stockouts in public facilities, and the increased availability of subsidised ACTs in the private sector.

## Introduction

The Affordable Medicines Facility- malaria (AMFm) was hailed as ‘one of the most important recent advances in fighting malaria’[1], designed to expand access to effective antimalarials in the public and private sectors[2]. An estimated 3.3 billion people are at risk of malaria, with 80% of cases in sub-Saharan Africa[3]. Artemisinin combination therapies (ACTs) are widely regarded as the best treatment for uncomplicated malaria[3]. However use remains low, reflecting both unreliable public sector supplies and low availability and high prices of ACTs in the private sector, which has an increasingly important role in treatment seeking for malaria[4]–[6]. These factors lead patients to use older, less effective antimalarials such as sulfadoxine-pyrimethamine (SP) and amodiaquine[4]. There is also concern about the use of oral artemisinin monotherapies, which may contribute to the development of artemisinin resistance[7].

The AMFm, an ACT subsidy mechanism, was set up by the Global Fund to Fight AIDS, Tuberculosis and Malaria to address some of these barriers to ACT access. AMFm was launched in 2010 as eight national scale programmes in seven countries, comprising Ghana, Kenya, Madagascar, Niger, Nigeria, Tanzania and Uganda (mainland Tanzania and Zanzibar were considered as separate pilots). AMFm aimed to increase availability, market share and use of quality-assured ACTs (QAACTs) while reducing prices, thereby increasing coverage[8]. This was also intended to result in ‘crowding out’ of other antimalarials from the market.

**Figure 1:**
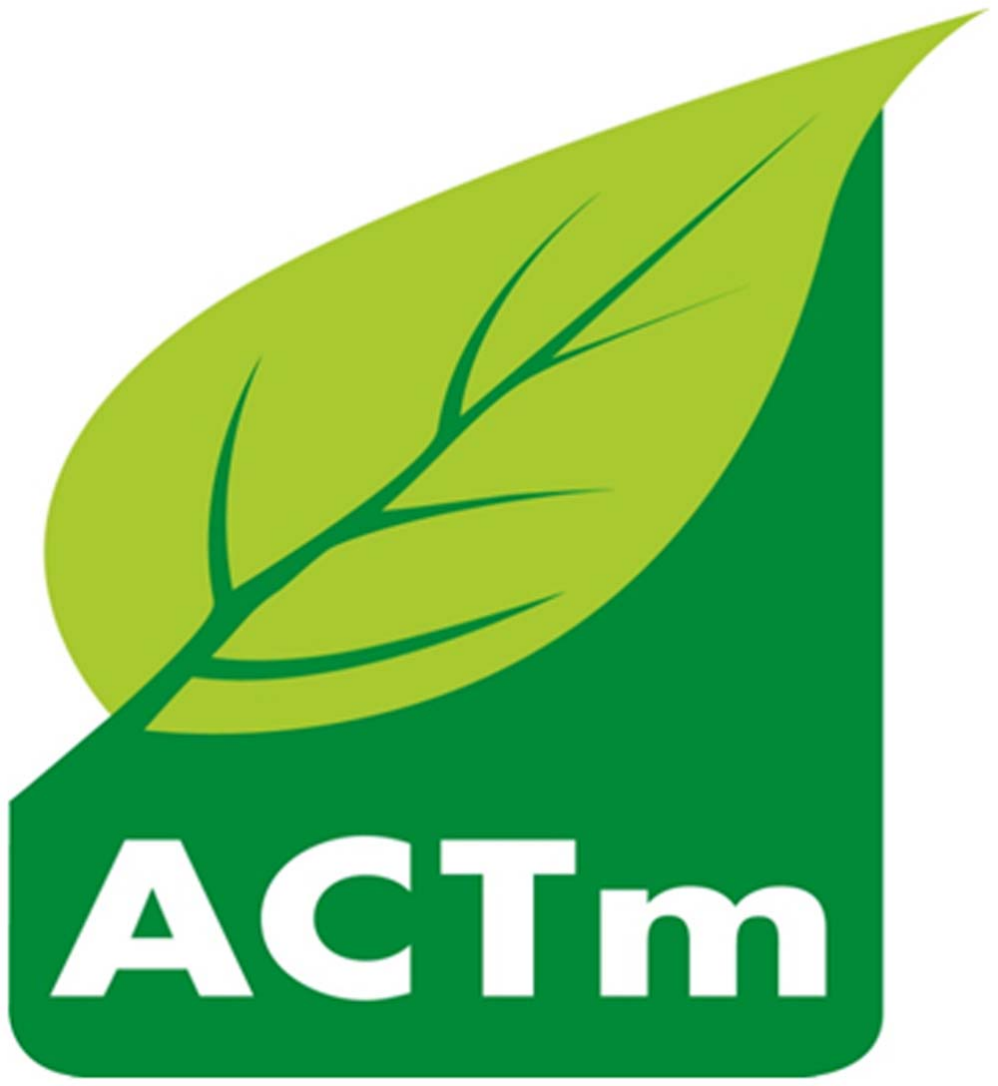
Green leaf logo.

AMFm consisted of three main elements: negotiations with QAACT manufacturers to reduce prices; a copayment made by the Global Fund to these manufacturers for every purchase made, representing 80–99% of the factory gate price; and supporting interventions to increase ACT awareness and appropriate use[9]. AMFm could operate through the public, private for-profit and private not-for-profit sectors. Eligible importers, termed first line buyers, placed orders with approved manufacturers. Orders were then forwarded to the Global Fund AMFm Secretariat for approval. All copaid QAACTs had a green leaf logo on the packaging for identification as a subsidised good quality antimalarial (Figure 1).

Despite these bold aims, AMFm has remained controversial, with concerns that the reduced price would still present access barriers for the very poor, that the subsidy would not be transmitted to the retail level and therefore not benefit the intended recipients, and that low levels of diagnostic coverage would lead to poor targeting and overtreatment of parasite negative individuals[10]–[12].

### Malaria treatment in mainland Tanzania

In Tanzania, about 90% of the population is at risk of malaria[13]. The Tanzanian government introduced the ACT artemether-lumefantrine (ALu) to replace SP as the first line drug for treatment of uncomplicated malaria in 2006, with quinine as second line treatment. According to national guidelines, ACTs are provided free at public facilities for under fives, pregnant women, the elderly and those who cannot afford to pay[14], although these exemptions are sometimes not fully implemented[15]. Oral artemisinin monotherapies have been banned since 2008, while non-oral artemisinin monotherapies are allowed for treatment of severe disease. ACTs are designated as prescription only medicines (POMs), while SP is over the counter (OTC). Pharmacies are allowed to stock POMs, while Duka la Dawa Baridi (DLDB, meaning ‘drug store’ in Kiswahili) are only allowed to stock OTC drugs. Since 2006, Tanzania has been in the process of upgrading DLDBs to Accredited Drug Dispensing Outlets (ADDOs), where dispensers undergo a 35 day training course and are allowed to dispense a limited range of POMs including ALu and quinine[16]. A few general stores and kiosks also stock antimalarials, although this is not permitted.

Diagnosis of fever cases in public health facilities was mainly based on symptoms alone until the Government changed the guidelines in 2010 to require parasitological confirmation for treatment of malaria. Malaria rapid diagnostic tests (mRDTs) were rolled out in public health facilities in Tanzania between 2010 and 2012[17], [18]. Drug retailers are not allowed to stock mRDTs.

### AMFm implementation

The AMFm grant agreement between the Global Fund and the Tanzanian government was signed in August 2010. By December 2011 ten private sector first line buyers were registered in mainland Tanzania, of which five had placed orders with manufacturers[19]. The first copaid drugs for the private for-profit sector arrived in October 2010. By the end of 2011, about 8 million ACT packs had arrived in country for this sector and by the end of 2012 an additional 16.6 million doses had been delivered. A maximum recommended retail price (RRP) of 1,000 Tanzanian Shillings (TSh) ($0.64) was set for an adult dose in private for-profit outlets[9].

The Medical Stores Department was registered as the first line buyer for the public sector. Public sector orders and deliveries were delayed, and only started arriving in July 2011. By December of that year 4.9 million doses had been delivered to the public sector, and by the end of 2012 a further 4.9 million doses. Additional public sector supplies were provided by the US President's Malaria Initiative, which donated 6.5 million ACT doses in 2011 and 4.7 million doses in 2012. Despite these additional supplies, high stockout levels in public health facilities were reported during AMFm implementation, although these were similar to stockout levels prior to AMFm[19].

AMFm supporting interventions began in January 2011 in mainland Tanzania, and included use of national level mass media and community level communications, to raise awareness of the copaid drugs, the RRP and the green leaf logo. The mass media campaign began after the national launch in April 2011, and included TV and radio adverts and printed material. Community level communications, including mobile video units, road shows, clinic shows and school activities were implemented through community health workers and community based organisations, and restricted to two districts per region due to budget constraints.

Training was the other major supporting intervention implemented in mainland Tanzania, focusing on the continued upgrading of drug stores to ADDOs. Upgrading had been occurring region by region since 2006. Prior to AMFm implementation, ADDOs had been introduced in eight of Tanzania's then 21 regions. Tanzania had 21 regions at the time of the study, though this has subsequently been increased to 25. An additional six regions were covered by the end of 2011, and a further six by the end of 2012, with progress slower than planned due to delays in disbursements from the Global Fund. In addition, a one day re-training programme covering malaria, Integrated Management of Childhood Illness and family planning was implemented in ADDOs in two regions in August to September 2011. Other smaller scale supporting interventions comprised strengthening of pharmacovigilance and monitoring and evaluation activities.

This paper explores the impact of AMFm from both the supply and demand sides, specifically assessing changes between baseline and endline (pre and post AMFm implementation) in the following five areas:

- Availability of QAACTs
- Price of QAACTs
- Market share of QAACTs
- Choice of provider for treatment of fever
- ACT use

Supply side data are drawn from outlet surveys conducted in 2010 and 2011 in all regions of mainland Tanzania as part of the multi-country Independent Evaluation of AMFm[19]. Demand side data are drawn from household surveys conducted in 2010 and 2012 in three regions of Tanzania with varying malaria transmission (Mwanza, Mbeya and Mtwara), as part of the IMPACT2 project (www.actconsortium.org/IMPACT2).

## Methods

The study had a non-experimental, before and after design. As AMFm was implemented nationally there were no comparison areas.

Outlet surveys were conducted using methods adapted from the ACTwatch project[20]. Baseline outlet survey data collection took place from September to November 2010, and endline data collection from October 2011 to January 2012. 49 wards were randomly selected at baseline and endline, respectively, with probability proportional to population size, stratified by urban/rural location. One ward had to be dropped at baseline so data from 48 wards was analysed. Wards were designated as urban or rural using National Bureau of Statistics Census classifications, with mixed wards classified as urban if more than 70% of the ward was classified as urban. In each selected ward, every outlet with the potential to sell antimalarials was visited, including public and private health facilities, pharmacies, ADDOs, drug stores, general stores, kiosks and community health workers. Outlets were identified using official lists from district and national authorities, by consulting with district pharmacists and other local leaders, and by driving or walking down every street within a ward to locate all outlets. In large wards with a population over 30,000 people, wards were segmented, and one or more segments of the ward were randomly selected for the survey. As pharmacies were relatively rare but thought to be a major source of treatment, they were oversampled, whereby all pharmacies in the district in which the selected ward was located were visited.

The sample size was calculated to detect a 20 percentage point change in outlets stocking a QAACT between baseline and endline, in urban and rural domains, with 80% power, 5% significance and at baseline assumed a design effect of 4 and 40% availability of QAACTs. Using these criteria, 305 outlets which stocked antimalarials were required in both urban and rural domains at baseline and endline. Estimates of the average numbers of outlets per ward were used to estimate the number of wards required to reach this number of outlets: 9 urban and 39 rural at baseline. This was then adjusted at endline to 20 urban and 29 rural wards, reflecting baseline estimates of availability and the higher degree of clustering observed at baseline in urban areas.

Screening criteria were used to identify outlets with an antimalarial in stock at the time of visit or within the previous three months. Following verbal consent, a questionnaire was conducted with the most senior staff member present, in Kiswahili, with data collected using Personal Digital Assistants (PDAs). Questions about outlet characteristics were asked, and details about every antimalarial in stock at the time of visit were recorded, as well as the volumes of each product sold in the past seven days.

Antimalarials were classified as quality-assured ACTs (QAACTs), non-quality-assured ACTs, artemisinin monotherapies (broken down by oral and non-oral forms), or non-artemisinin therapies such as SP, chloroquine, mefloquine and amodiaquine. QAACTs are ACTs that comply with the Global Fund's quality assurance policy[21]. Price and market share data were calculated using Adult Equivalent Treatment Doses (AETDs), the amount of a drug needed to completely treat a 60 kg adult[22]. For example, to calculate the price per AETD of a paediatric package of ALu with 6 standard tablets (20 mg artemether and 120 mg lumefantrine), the price would be multiplied by 4 to calculate what it would cost for an adult equivalent dose of 24 tablets.

The household surveys took place in Mwanza, Mbeya and Mtwara regions, which have varying malaria transmission and epidemiology. Baseline household data collection took place from June to October 2010, and endline from May to September 2012. Mwanza is located next to Lake Victoria, Mtwara is in the south on the coast, and Mbeya is located in the southern highlands. In 2011–2012 malaria prevalence among children aged 6–59 months was 18.6% in Mwanza, 17.4% in Mtwara and 0.5% in Mbeya[23]. Based on the national distribution of socio-economic status, Mbeya had the lowest percentage of people living in the lowest wealth quintile of the three regions, at 7.7%, compared to 20.8% in Mwanza and 35.5% in Mtwara [24]. mRDT roll out in public facilities took place in all three regions between the baseline and endline household surveys, in early 2011 in Mwanza and Mbeya, and in mid-2012 in Mtwara. The ADDO programme had been implemented in Mbeya and Mtwara regions by baseline, and was implemented in Mwanza region after endline household data collection.

In each region, 80 enumeration areas (EAs) were randomly selected using probability proportional to population size. In selected EAs, every household was visited and mapped using Global Positioning System. After obtaining a list of all the households within an EA, 32 households were randomly selected while in the field, 24 to be visited first, and the remaining eight to be included sequentially as replacements if any of the initial 24 households were unavailable or refused to be interviewed. At baseline, the maximum number of households visited per enumeration area was 32 in Mwanza, and 24 in Mbeya and Mtwara. At endline the maximum was 24 in all regions. The sample size was calculated to detect a 10 percentage point increase in children under five with a febrile illness who obtained ACT within 24 hours of fever onset. With 80% power, 5% significance and an assumed design effect of two, 480 children under five with fever were required in each region.

The questionnaire was translated into Kiswahili, and data were collected using PDAs. Questions on household demographics were asked of the household head, and questions on history of fever were asked of all household members. All members who reported fever in the 14 days prior to interview were asked about treatment sought and drugs and blood tests obtained. The guardian was interviewed on behalf of children below 12 years old. In addition, a finger prick blood sample was taken from all consenting members, from which an mRDT was performed (ICT Diagnostics, Cape Town, South Africa). Written consent was obtained from everybody who was interviewed or had blood taken, or from their guardian.

Data analysis for the outlet and household surveys was performed using Stata version 11 (College Station, Texas). Stata survey procedures were used to account for the survey design and stratification. Changes in availability and use were assessed using the design based F-test. R version 2.14.2 was used in outlet survey analysis for obtaining p-values for the change in retail prices using the Wilcoxon Rank Sum test. 2011 and 2012 prices were converted to 2010 prices using the Tanzania consumer price index. Prices were converted to US dollars using the average interbank rate for 2010. Socio-economic status quintiles were calculated using principal components analysis, based on the first principal component, and using standard Demographic and Health Survey variables[24].

In addition, key informant interviews were conducted at national, regional and district level to capture information on the process of AMFm implementation and relevant contextual factors[19]. Interviewees were drawn from government bodies such as the National Malaria Control Programme and the Tanzania Food and Drug Authority, non-governmental organisations such as the Clinton Health Access Initiative, organisations implementing supporting interventions, as well as regional and district medical officers and other local staff. We draw on the information gathered to inform the Discussion section of this paper.

Ethical approval was obtained from the Institutional Review Board of Ifakara Health Institute and the London School of Hygiene and Tropical Medicine Research Ethics Committee for both the outlet and household surveys, including collection of blood samples during the household survey, and also from the Institutional Review Board of ICF International for the outlet survey. The investigator from the US Centers for Disease Control and Prevention provided technical assistance for the household survey but was not actively engaged in data collection for either survey.

## Results

### Antimalarial availability, price and market share

Of all outlets visited, 709 met the screening criteria at baseline and 799 at endline (Table 1). 93.0% and 99.9% of outlets that met the screening criteria were interviewed at baseline and endline respectively.

**Table 1:** Description of outlet survey sample at baseline and endline.

	Baseline					
	Number enumerated	Number screened	Number which met screening criteria*	Number interviewed	Number with antimalarials in stock at time of visit	Number of antimalarials audited
Public Health Facilities	76	72	70	64	61	232
Private Health Facilities	40	40	35	34	33	191
Specialised Drug Sellers	545	524	502	467	463	5,020
General Retailers	2,484	2,478	199	92	72	90
Community Health Workers	5	5	2	2	1	3
**Total**	**3,150**	**3,119**	**709**	**659**	**630**	**5,536**
	Endline					
Public Health Facilities	62	59	59	59	55	244
Private Health Facilities	37	34	32	32	32	275
Specialised Drug Sellers	743	687	684	684	683	9,132
General Retailers	2,936	2,921	24	23	17	40
Community Health Workers	1	1	0	0	0	0
**Total**	**3,779**	**3,702**	**799**	**798**	**787**	**9,691**

*An outlet met the screening criteria if there was an antimalarial in stock at the time of visit or within the previous three months.

Source: Outlet surveys in 2010 and 2011.

Outlets were classified as: public health facilities (dispensaries, health centres and hospitals); private health facilities (for-profit and not-for-profit dispensaries, clinics and hospitals); specialised drug sellers (SDSs) (pharmacies and drug stores, including DLDB and ADDOs); and general retailers (general stores and kiosks). Private not-for-profit and private-for-profit health facilities were pooled due to low numbers obtained. Only one community health worker stocked an antimalarial at baseline and none at endline, so results for this subgroup are not presented separately but are included in total estimates.

In total, 15.8% and 14.0% of outlets visited stocked an antimalarial at baseline and endline, respectively (p = 0.31). Antimalarial availability was over 80% at baseline and endline in public health facilities, private health facilities and SDSs, while general retailers had the lowest availability of 4.3% at baseline and 0.9% at endline (p = 0.024) Of outlets stocking antimalarials, the percentage with at least one staff member with a health related qualification was above 98% in public and private health facilities at both baseline and endline, and increased from 89.7% to 97.1% in SDSs (p = 0.002).

#### Availability of QAACTs

**Figure 2:**
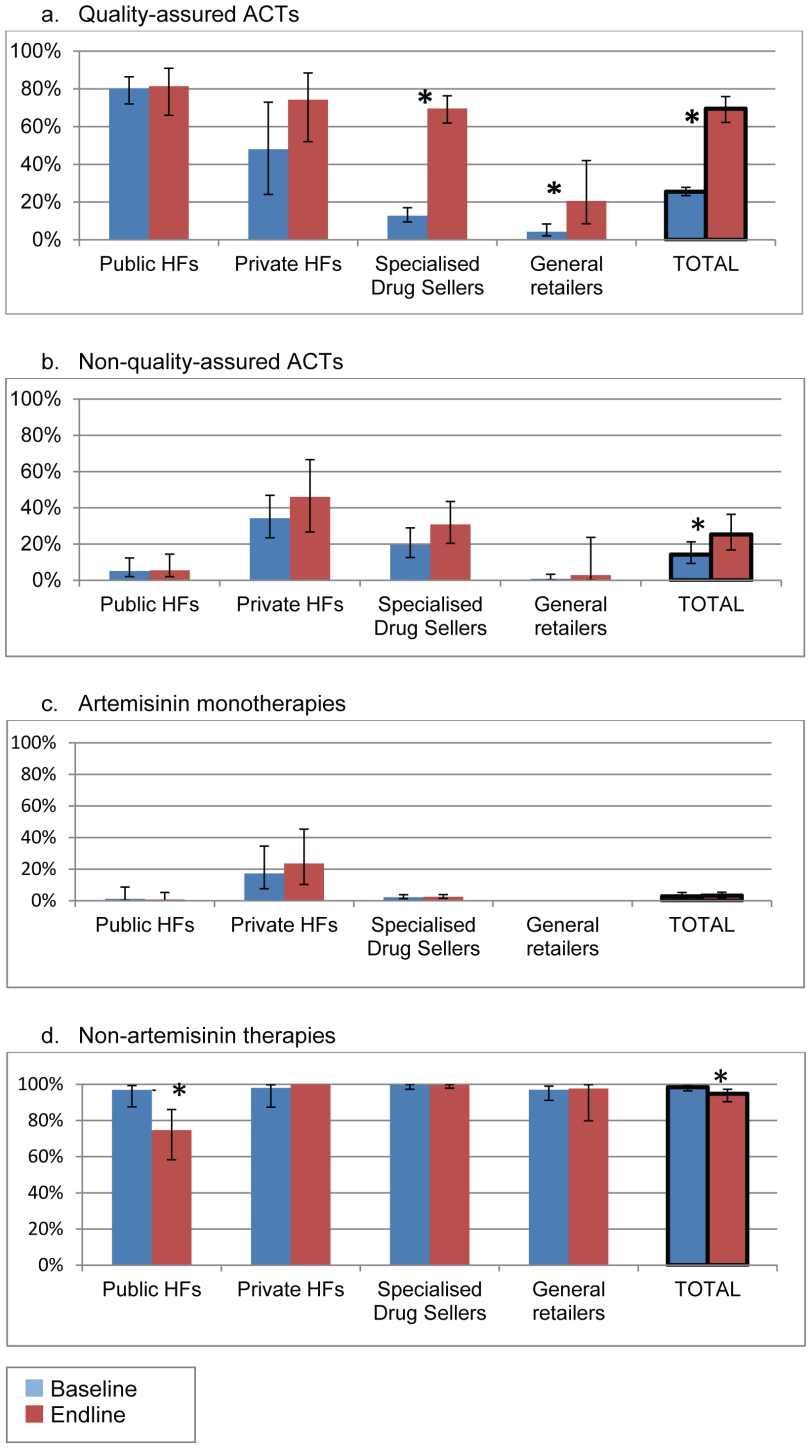
Of all outlets stocking antimalarials, percentage stocking (a) quality-assured ACTs, (b) non-quality-assured ACTs, (c) artemisinin monotherapies, and (d) non-artemisinin therapies, by outlet type at baseline and endline. * denotes p<0.05 for change over time Error bars denote 95% confidence intervals HFs: Health facilities Source: Outlet surveys in 2010 and 2011.

Availability of specific antimalarial categories was calculated out of outlets stocking any antimalarials. QAACT availability increased from 25.5% among all outlets at baseline to 69.5% at endline (p<0.001) (Figure 2). This was mainly due to the substantial increase in SDSs, where availability increased from 12.8% to 69.6% (p<0.001). Availability in general retailers increased from 4.3% to 20.6% (p<0.001). No change was seen in QAACT availability in public health facilities, which was 80.1% at baseline and 81.4% at endline (p = 0.86). A decrease in the availability of non-artemisinin therapies in public health facilities led to a small but significant decrease overall (98.4% to 94.8%, p = 0.020). There was a small increase in availability of non-quality-assured ACTs (14.2% to 25.3%, p = 0.046), which was not significant in any one outlet type. The first line drug ALu accounted for 94.8% of QAACTs and 4.7% of non-quality-assured ACTs found in stock at baseline, and 94.4% of QAACTs and 9.0% of non-quality-assured ACTs at endline.

#### Price of QAACTs

The median price of QAACTs was zero in public and private facilities as they were supposed to be provided free in all public and most private not-for-profit facilities. Median price data for SDSs are shown in Table 2, for tablets only to enhance comparability across antimalarial types as QAACTs are only available in tablet form. The median price of QAACT tablets in SDSs fell from $5.63 to $0.94 (p<0.001). Non-artemisinin therapy tablets had a median price in SDSs below $1 at baseline and endline, and at endline the median price of QAACT tablets was equal to that of non-artemisinin therapy tablets. The median price of non-quality-assured ACT tablets also decreased in SDSs from $7.92 to $6.87 (p = 0.025) though the magnitude of the decrease was not as great as that for QAACTs.

**Table 2:** Median retail price per Adult Equivalent Treatment Dose of antimalarial drugs in tablet form in specialised drug sellers at baseline and endline (2010 USD).

	Baseline		Endline	
	N	Median price [IQR]	N	Median price [IQR]
**Quality-assured ACTs**	277	5.63 [1.70–8.45]	1,795	0.94 [0.62–1.25]*
**Non-quality-assured ACTs**	1,130	7.92 [5.99–13.52]	1,781	6.87 [3.75–12.96]
**Non-artemisinin therapies**	1,960	0.85 [0.63–1.27]	2,719	0.94 [0.63–1.25]

IQR: Inter quartile range.

*denotes p<0.05 for change over time using Wilcoxon rank sum test.

Artemisinin monotherapies not presented due to low numbers obtained.

Source: Outlet surveys in 2010 and 2011.

#### Market share of QAACTs

**Figure 3:**
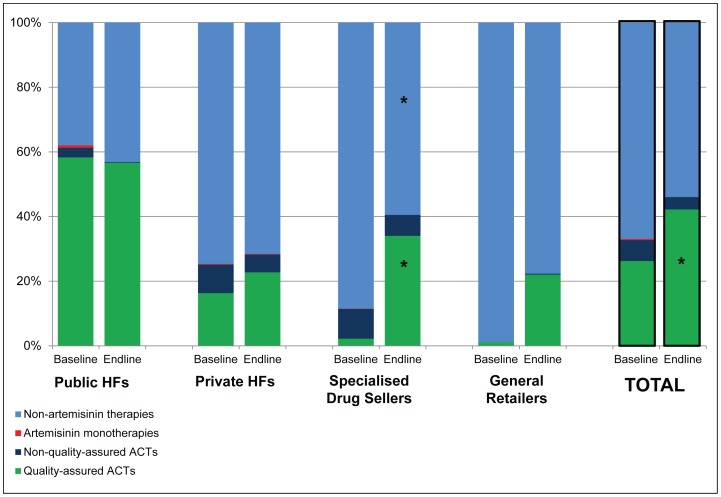
Market share by antimalarial category: percent distribution of antimalarial sales volumes by antimalarial category at baseline and endline. HFs: Health facilities *denotes p<0.05 for change over time Source: Outlet surveys in 2010 and 2011.

The market share of QAACTs as a percentage of all reported antimalarial sales increased from 26.2% to 42.2% among all outlet types combined (p = 0.033) (Figure 3), with a particularly large increase in SDSs from 2.2% to 34.0% (p<0.001). No significant changes were seen in QAACT market share in public or private health facilities. The market share of non-quality-assured ACTs did not change significantly and the market share of artemisinin monotherapies was minimal, below 1.0% in all outlet types at baseline and endline. Therefore the increased QAACT market share was at the expense of non-artemisinin therapies, for which the market share decreased by 13 percentage points among all outlet types combined.

#### Urban/rural variation in availability, price and market share

When broken down by urban and rural areas, large increases were seen in QAACT availability in both urban and rural areas overall, and especially in SDSs (Annex S1). At baseline, there was higher availability of QAACTs in urban SDSs than rural SDSs (p = 0.028), but at endline there was no significant difference between the areas. Decreases in median QAACT prices were seen in both urban and rural areas, although greater reductions were seen in urban areas as there were few QAACTs in rural areas at baseline and the majority of these were very inexpensive (Annex S2). The increase in QAACT market share was almost completely due to changes in rural areas, where market share increased by 25.4 percentage points, compared with 1.8 percentage points in urban areas (Annex S3). However, significant increases were seen in market share in SDSs in both urban and rural areas.

### Treatment sought and obtained for malaria

5,423 and 5,511 households were interviewed at baseline and endline, respectively, involving 20,874 and 20,102 full interviews of household members or their guardian (Table 3). Over four fifths of household heads worked in agriculture, and about half had completed primary school. Overall parasite prevalence among all age groups according to study mRDT results was 17.5% at baseline and 12.0% at endline (p<0.001).

**Table 3:** Description of household survey sample at baseline and endline.

	Baseline			Endline		
**Description of households (HHs):**						
HHs mapped	31,600			39,864		
HHs selected	6,177			6,051		
HHs that participated	5,423			5,511		
**Description of household members:**	Age group			Age group		
	<5	≥5	Total	<5	≥5	Total
HH members registered			26,924			25,407
HH members interviewed	4,143	16,731	20,874	4,063	16,034	20,102
Percentage interviewed who were male	49.1	41.3	42.9	49.6	41.5	43.2
HH members with study mRDT results*	3,986	15,940	19,926	3,732	14,384	18,116
Percentage parasite positive by study mRDT	15.9	17.8	17.5	11.7	12.1	12.0
**Socio-demographic characteristics of households:**						
**Occupation of household head:**	**% (95% CI)**			**% (95% CI)**		
Agriculture	82.1 (77.9–85.7)			82.6 (79.1–85.6)		
Unskilled manual labour	4.4 (3.2–6.0)			3.7 (2.9–4.8)		
Skilled manual labour	2.9 (2.1–3.9)			2.2 (1.7–2.8)		
Domestic service	1.7 (1.0–2.8)			1.4 (1.0–2.0)		
Sales and services	5.7 (4.3–7.8)			5.9 (4.4–7.8)		
Clerical	0.2 (0.0–0.6)			0.0 (0.0–0.3)		
Professional/technical/managerial	3.0 (0.2–3.8)			4.2 (3.1–5.7)		
**Education status of household head:**						
None	26.0 (23.9–28.2)			28.5 (26.4–30.7)		
Primary Incomplete	15.8 (14.5–17.2)			15.4 (13.9–16.9)		
Completed Primary	51.9 (49.7–54.1)			50.0 (48.0–52.1)		
Completed 4 years of secondary or higher	6.3 (4.9–8.0)			6.1 (4.8–7.7)		

*Malaria Rapid Diagnostic Test results not obtained due to people not consenting to the test, leaving before the test was conducted or unreadable results.

Source: Household surveys in 2010 and 2012.

#### Choice of provider for treatment of fever

Overall 69.5% of people with fever sought care at baseline and 73.6% at endline (p = 0.074), with seeking care defined to include care sought outside the home and drugs obtained from home/a neighbour. Only 3.8% of people with fever at baseline and 2.7% at endline sought care in more than one place.

**Figure 4:**
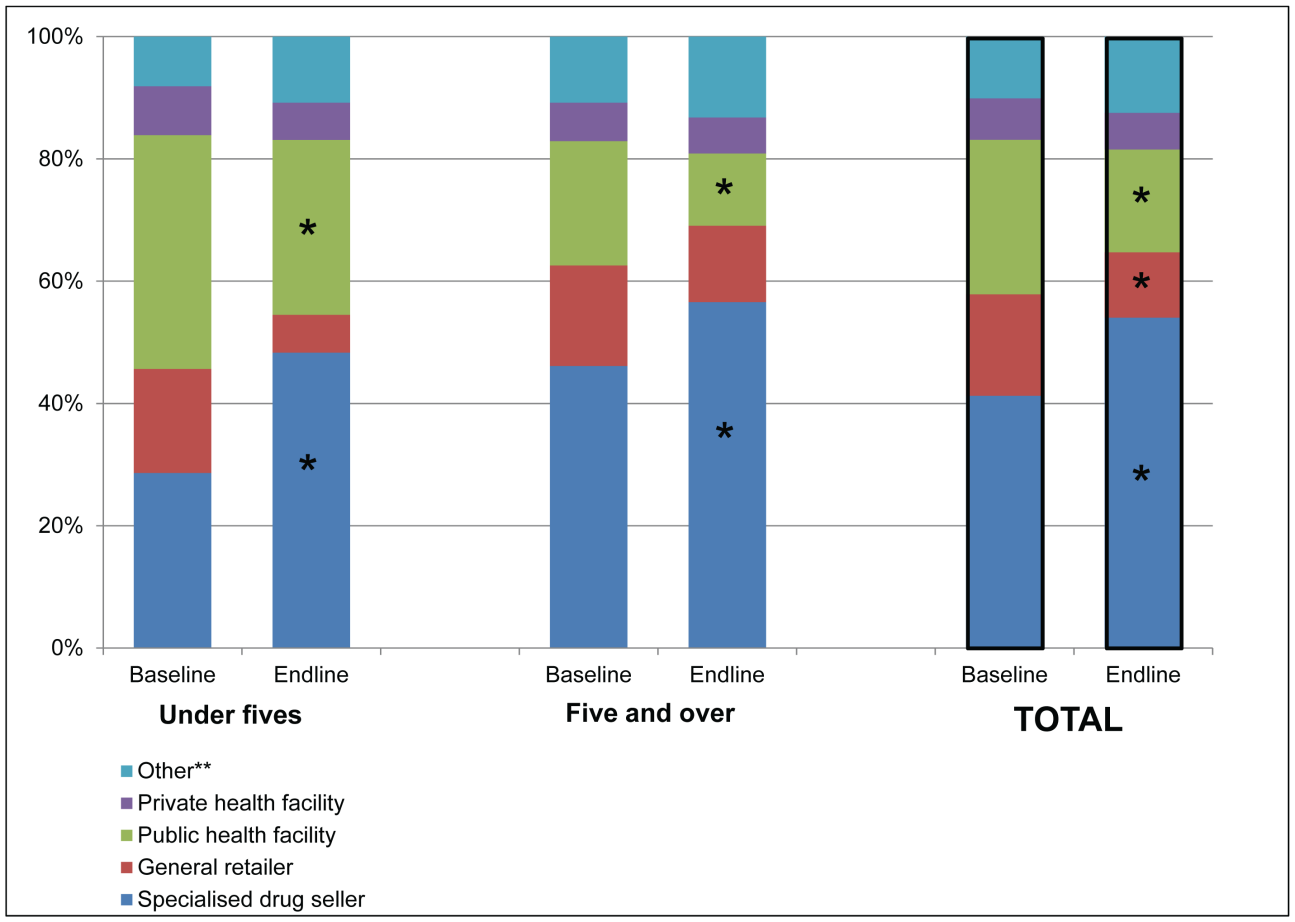
Choice of first provider for treatment of fever in the past two weeks by age group at baseline and endline. * denotes p<0.05 for change over time **Other includes seeking treatment from home, a neighbour or a traditional healer Source: Household surveys in 2010 and 2012.

There was a marked shift from baseline to endline away from public health facilities and general retailers and towards SDSs overall and in each age group, with an increase from 41.3% to 54.1% in people visiting SDSs (p<0.001) and a decrease from 25.3% to 16.8% in people visiting a public health facility (p<0.001) as the first source of care overall (Figure 4). The change in use of SDSs and public health facilities was considerable in both age groups, with the increase in SDS use being highest among under fives, from 28.6% to 48.3% (p<0.001).

#### ACT use

**Figure 5:**
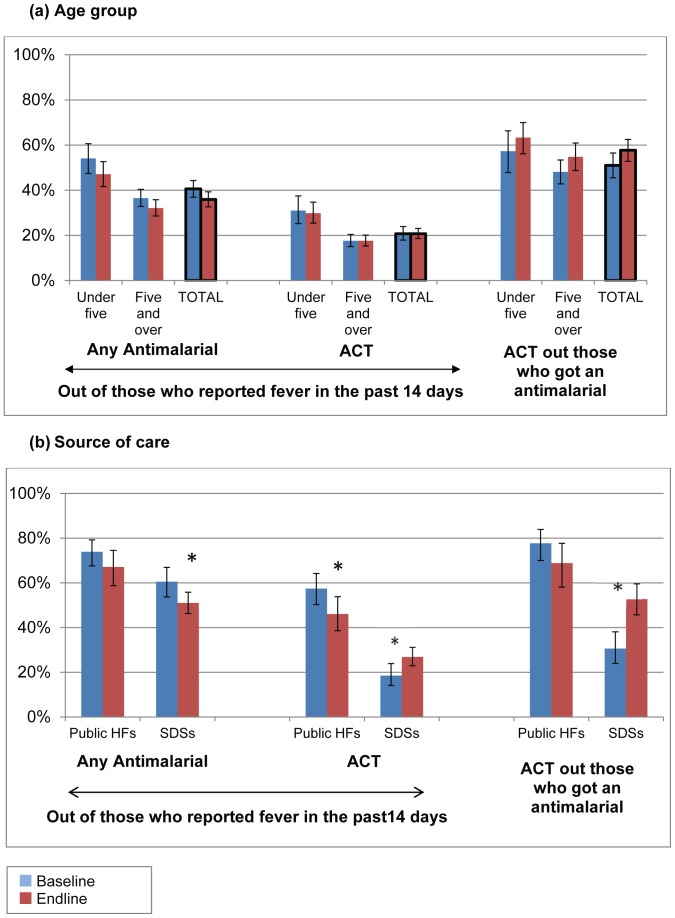
Percentage of people who reported fever in the past two weeks who obtained any antimalarial and an ACT at baseline and endline by (a) age in years, and (b) source of care. SDSs: Specialised Drug Sellers; Public HFs: Public health facilities * denotes p<0.05 for change over time; Error bars denote 95% confidence intervals Source: Household surveys in 2010 and 2012.

Overall the percentage of people with fever who obtained an antimalarial was 40.6% at baseline and 35.9% at endline (p = 0.070), and did not change to an important degree in either age group (Figure 5a). The percentage of people with fever who obtained an ACT was 20.7% at both baseline and endline (p = 1.00). There was weak evidence for an increase in the percentage of people who obtained an ACT, out of those who obtained an antimalarial between baseline (51.0%) and endline (57.7%) (p = 0.077), corresponding with the slight fall in all people with a febrile illness getting an antimalarial and no change in those getting an ACT.

**Figure 6:**
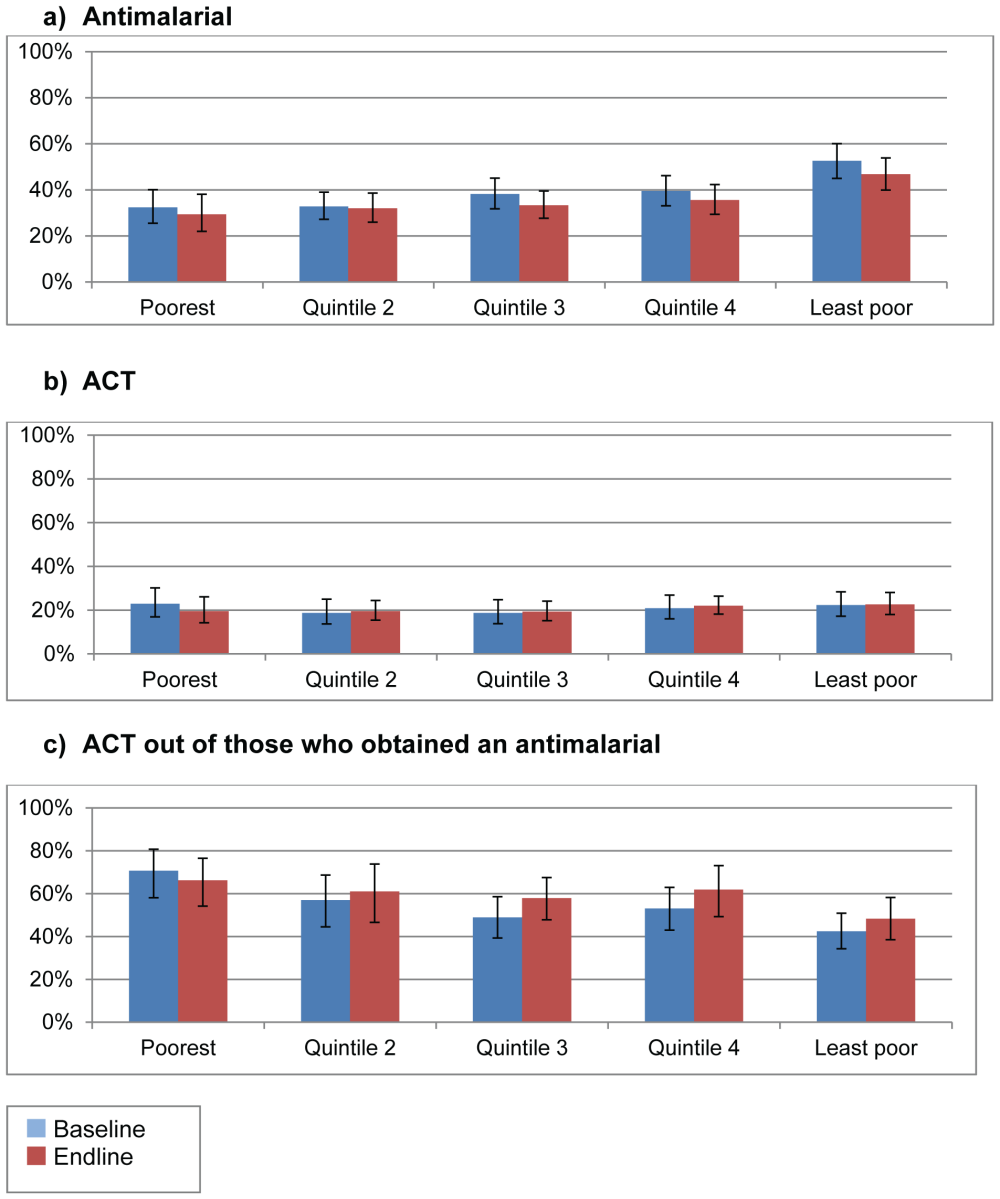
Treatment obtained for fever in the past two weeks by socio-economic status. * denotes p<0.05 for change over time Error bars denote 95% confidence intervals Source: Household surveys in 2010 and 2012.

Other marked changes were seen in these three core household indicators when broken down by where the patient sought care. Figure 5b shows the data separately for visits to public health facilities and SDSs, the two sources where the majority of people sought care. In public health facilities the percentage of people obtaining an ACT decreased from 57.4% to 46.1% (p = 0.035), but there were no marked differences in the percentage of people obtaining an antimalarial, or an ACT out of those obtaining an antimalarial. The percentage of people visiting SDSs obtaining an antimalarial decreased from 60.5% to 51.0% (p = 0.026), while the proportion obtaining an ACT increased from 18.5% to 26.9% in these outlets (p = 0.015). The percentage obtaining an ACT out of those who obtained an antimalarial increased from 30.6% to 52.7% in SDSs (p<0.001). When broken down by socio-economic status there was no substantial changes in any of the three use indicators in any quintile (Figure 6).

#### Use of diagnostic tests

The percentage of people obtaining a blood test was 17.6% at baseline and 21.4% at endline overall (p = 0.78), with no major change in any age group. In public health facilities the percentage of people who obtained a blood test increased from 28.7% to 46.6% (p<0.001), while in SDSs there was weak evidence of a decrease (4.1% to 2.1%, p = 0.061)

#### Price paid for ACTs

Households were also asked about the price they had paid for antimalarials. The median price paid for ALu tablets from SDSs at baseline was $1.41 (interquartile range (IQR) $0.85 to $1.88) per AETD, and at endline was $1.29 (IQR $0.86 to $2.15). At endline, the median price of ALu with the green leaf logo from SDSs was $1.08 (IQR $0.81 to $2.15) and without the logo was $1.29 (IQR $1.08 to $2.15). At baseline 68% of ACTs from public health facilities were reported to have been obtained for free and 63% at endline (p = 0.53). Of drugs not obtained for free, the median cost was $0.70 at baseline and $1.08 at endline in this sector.

#### Regional and urban/rural variation in provider choice and ACT use

Somewhat different treatment seeking patterns were seen in rural and urban areas and across the three regions visited (Annex S4–S6). In rural areas and in Mbeya and Mtwara regions a shift away from public health facilities towards SDSs occurred, but in Mwanza and in urban areas this was not significant, reflecting the high usage of SDSs at baseline. The proportion of people with febrile illness who obtained an antimalarial decreased significantly in Mtwara, and in rural areas, while the proportion of people obtaining ACT and the proportion obtaining ACT out of those obtaining an antimalarial did not change to a substantial degree in any setting.

## Discussion

This paper has presented results from large scale baseline and endline outlet and household surveys to explore the impact of AMFm from both a supply and demand perspective. The paper demonstrates how very different inferences can be drawn from looking at each side of the market, and therefore the importance of a holistic approach to evaluation.

Before discussing the results further it should be noted that the surveys had several limitations. They were both based on reported data from providers and community members, and therefore potentially subject to recall bias. In addition, outlet staff may have biased their answers to make them more socially desirable or due to fear of regulatory consequences. For example, they may have concealed certain POMs, including ACTs, if they were concerned that their outlet was not authorised to sell them, especially at baseline before ACTs were widely promoted in the private sector. In addition, they may have stated lower retail prices than they actually charge, especially at endline when the RRP was widely publicised. Similarly, in the household survey, respondents may have overstated their use of ACTs if they knew this was the ‘correct’ answer, or stated that they went to a public health facility instead of another source.

Another factor to consider is the different timings and geographical coverage of the surveys. The outlet surveys were conducted between September 2010 and January 2012, in all regions of mainland Tanzania, while the household surveys were conducted between June 2010 and September 2012, in three regions only. The endline outlet survey was conducted relatively early on during AMFm implementation: twelve months after subsidised drugs first arrived and five months after the communication campaigns commenced, while the household survey was conducted nine months later. It is unclear whether a longer time period before endline would be associated with greater impact as the programme became more established, or less impact as the effect of initial training and communications waned.

Finally, the evaluation was based on a before and after study design. Lack of control areas means that it is challenging to assess what would have happened in the absence of AMFm and to what extent the changes seen are attributable to the programme. Below we draw on findings from key informant interviewers to address these issues where possible.

Key informants indicated that there were no major delays in obtaining copaid drugs by private sector buyers and the supply chain from manufacturer to buyers generally operated smoothly, after an initial slow start in early 2011. The outlet surveys showed that in the private sector QAACT availability and market share increased while retail price decreased after AMFm implementation, with the household surveys showing large improvements in ACT use among SDS customers. In these outlets, although the proportion of people getting an antimalarial decreased, those obtaining an ACT increased, resulting in a substantial increase in the proportion of people getting an ACT out of those getting an antimalarial. Key informants did not identify any other projects or contextual issues that could have been responsible for changes of this scale in the retail sector, implying that they were very likely due to AMFm.

By contrast, outlet survey data showed no significant change was seen in QAACT availability or market share in the public sector. Household survey data showed that the proportion of people obtaining an ACT at public facilities actually fell. This likely partly reflected the persistence of ACT stockouts in public facilities, and the significant increase in blood tests between baseline and endline due to mRDT roll out. Health facility surveys in the same three regions in 2010 and 2012 also reported a significant decrease in the percentage of people with fever obtaining ACTs from 39.9% to 21.3%, along with an increase in the percentage of people obtaining a blood test from 15.9% to 55.8%[18].

Key informants reported that the supply chain in this sector proved more problematic than that for the private sector, with severe delays in procuring and obtaining copaid drugs through AMFm channels. The 4.9 million doses of copaid QAACTs delivered by the end of 2011 were estimated to be only two to four months worth of supplies, and the extra doses donated by the US President's Malaria Initiative were still insufficient to prevent stockouts due to the low stock levels before AMFm, leading to similar QAACT availability in public health facilities at baseline and endline. These delays in receiving public sector copaid drugs were said to have been partly caused by delays in ordering due to the confusion over the new grant mechanisms, delays in approvals being granted, and delays in delivery owing to manufacturers' inability to meet the high global demand for drugs at the time.

Despite the improvements seen on the supply side in the private sector, household survey data showed that the overall proportion of people obtaining an ACT during a febrile illness had not changed, with only one fifth of patients obtaining an ACT before and after AMFm implementation. There are two possible explanations for this apparent paradox. First, while ACT use increased in the private sector, there was a reduction in the proportion of public sector patients obtaining ACT, reflecting increased use of mRDTs, as described above. Secondly, the proportion of people obtaining an ACT remained higher in the public sector than the private sector at endline, so the shift in treatment seeking towards the private sector therefore also had a negative effect on the overall proportion of people getting ACTs. These two effects therefore cancelled out the increase in ACT use in the private sector. To clarify further, at baseline, 25% of visits for fever/malaria treatment were to the public sector, of which 57% resulted in an ACT being obtained, compared to 41% and 19% respectively in SDSs; at endline 17% of visits were to the public sector and 46% of these obtained an ACT, while this was 54% and 27% respectively in SDSs. The result was that at baseline, of people with fever, 14% visited a public health facility and obtained an ACT, while 8% visited an SDS and obtained an ACT; at endline, 8% of people visited a public health facility and obtained an ACT, and 15% visited an SDS and obtained an ACT. Overall, the proportion of people who visited a public health facility or drug store and obtained an ACT therefore remained unchanged at 22%.

The market share of QAACTs overall increased significantly while the use of ACTs did not change, which could be affected by certain factors such as the different timings and geographical areas of the outlet and household surveys. At baseline the QAACT market share could potentially have been underreported which would have resulted in an overestimate of the change in market share.

The observed shift in treatment seeking behaviour may have reflected a number of factors. Qualitative data collected as part of the IMPACT2 project indicated that community members were very aware of public sector drug stockouts, and as a result tended to bypass public facilities and seek treatment elsewhere[25]. Coupled with an increasingly abundant supply of good quality, affordable medicines in the retail sector due to AMFm this could have encouraged people to make the private sector their first source of care. A continuation of the ongoing general expansion of the private health sector over time could also have played a part, although the shift seen over one year is likely too large to be attributed to this trend alone. The upgrading of DLDB to ADDOs might also be expected to increase the number of outlets selling POMs and therefore ACTs, encouraging more people to use the private sector. However, the shift in treatment seeking was seen in both ADDO and non-ADDO regions, and DLDB were also known to widely stock POMs illegally.

Household survey data showed no change in the price paid for ALu tablets, as the prices paid at baseline were very low, and much cheaper than the QAACT prices reported in the outlet survey at baseline. A possible explanation for this is that some ALu tablets in SDSs at baseline may have been leaked from public health facilities, and sold very cheaply to household members, but concealed from interviewers during the baseline outlet survey. It is also likely that relatively costly ALu tablets available in the private sector at baseline were too expensive for most community members, and therefore rarely purchased or recorded in the household survey.

A study in relatively remote areas of two Tanzanian regions in 2011–12 showed similar outlet survey results, with availability of AMFm subsidised ACTs increasing from 25% to 88% in ADDOs in Mtwara, and from 3% to 62% in Rukwa between February 2011 and January 2012 [26]. This was accompanied by a decrease in median price from $1.03 to $0.81. However, household surveys in the same region found a significant increase in ACT use among suspected malaria cases from 54.6% to 67.8%, mainly reflecting increased ACT use in the retail sector[27]. A shift in treatment seeking towards the retail sector was seen, but in contrast to our findings, this reflected a shift from those not seeking care rather than from the public sector. However, results from the Tanzania HIV/AIDS and Malaria Indicator Surveys in 2010 and 2012 were consistent with our study findings, showing no change in the proportion of children under five who obtained an ACT out of all those with fever, or out of those who obtained an antimalarial [23].

In comparison with the other seven AMFm pilots, mainland Tanzania was fairly successful in terms of outlet survey outcomes [9], with the third highest increase in QAACT availability, and the third largest fall in median QAACT price, but only the sixth highest increase in overall QAACT market share.

Baseline and endline household survey data were available for four of the other seven AMFm pilots. Similar to mainland Tanzania, results from Zanzibar also showed large improvements in QAACT availability, affordability and market share in the private for-profit sector but no change in overall ACT use[28]. As above, this likely reflected a reduction in the relative importance of the public sector as a source of antimalarials, with the share of all antimalarials distributed through the public sector falling from 37% at baseline to 13% at endline[19]. However, these contrasting household and outlet survey results were not found in Uganda, Nigeria or Madagascar. Uganda experienced a large and significant increase in ACT use in under fives (24.0 percentage points)[28], despite weaker performance on outlet survey outcomes. Nigeria and Madagascar saw smaller increases in ACT use of 6.7 and 5.0 percentage points respectively. Household survey data were not available for Kenya and Ghana, which experienced the strongest results for outlet survey indicators.

The Global Fund has decided to integrate AMFm into core Global Fund grant management, after a transition period in 2013[29]. This means that eligible countries will be able to use their core Global Fund resources to buy copaid drugs under the AMFm system, but this use of grant funds for ACT subsidies will compete with all other interventions for malaria, HIV/AIDS and tuberculosis. The Tanzanian government has committed to continuing with ACT subsidies, though concerns have been raised about poor targeting of copaid ACTs to those with malaria in the private sector. For example, Briggs *et al.* showed that after AMFm implementation in Mwanza and Mtwara, only 20% of people purchasing ACTs from DLDBs or ADDOs were parasitaemic[30]. Strategies to increase diagnostic use in the private sector have received considerable attention in national and international policy discussions[31], with some stakeholders keen to build on experience from Cambodia where subsidised mRDTs have been distributed through the private sector for over a decade[32]. The Tanzanian government is currently piloting the introduction of low cost and subsidised mRDTs in ADDOs, with the intention of improving targeting of subsidised ACTs to those with confirmed parasitaemia.

This research has highlighted that other key policy considerations should include how to combine improved access to quality, affordable ACTs in the private sector while maintaining use of the public sector, particularly through improvements in public sector ACT availability. Ultimately the goal should be to have affordable, high quality testing and treatment available in both the public and private sectors.

## Supporting Information

Annex S1 **Of all outlets stocking antimalarials, percentage stocking (a) quality-assured ACTS, (b) non-quality-assured ACTs, (c) artemisinin monotherapies, and (d) non-artemisinin therapies, at baseline and endline by urban and rural areas.**
(DOCX)

Annex S2 **Median retail price per Adult Equivalent Treatment Dose of antimalarial drugs in tablet form in specialised drug sellers at baseline and endline by urban and rural areas (2010 USD).**
(DOCX)

Annex S3 **Market share by antimalarial category: percent distribution of antimalarial sales volumes by antimalarial category at baseline and endline by rural and urban areas.**
(DOCX)

Annex S4 **Description of household survey sample at baseline and endline by region and urban and rural areas.**
(DOCX)

Annex S5 **First treatment sought for febrile illness at baseline and endline, by region and urban and rural areas.**
(DOCX)

Annex S6 **Treatment obtained for malaria at baseline and endline, by region and urban and rural areas.**
(DOCX)
