# Supplementary material for: Has Tanzania Embraced the Green Leaf? Results from Outlet and Household Surveys before and after Implementation of the Affordable Medicines Facility -Malaria
Source: PLoS One. 2014 May 9;9(5):e95607. doi: 10.1371/journal.pone.0095607 (PMC4015933; doi:10.1371/journal.pone.0095607)
Supplement: Annex S1 — Of all outlets stocking antimalarials, percentage stocking (a) quality-assured ACTS, (b) non-quality-assured ACTs, (c) artemisinin monotherapies, and (d) non-artemisinin therapies, at baseline and endline by urban and rural areas. (DOCX) [file pone.0095607.s001.docx]

**Annex S1:** Of all outlets stocking antimalarials, percentage stocking (a) quality-assured ACTs, (b) non-quality-assured ACTs, (c) artemisinin monotherapies, and (d) non-artemisinin therapies, at baseline and endline by urban and rural areas

|  | **URBAN** | | **RURAL** | |
| --- | --- | --- | --- | --- |
|  | **Baseline** | **Endline** | **Baseline** | **Endline** |
|  | **Quality-assured ACTs** | | | |
| Public Health Facilities | 86.5 (72.6-93.9) | 100* | 79.6 (70.5-86.5) | 80.6 (64.2–90.6) |
| Private Health Facilities | 44.2 (15.4-77.4) | 74.0 (50.7-88.7) | 49.5 (19.7-79.6) | 74.5 (28.1-95.6) |
| Specialised Drug Sellers | 19.1 (13.0-27.0) | 70.7 (60.6-79.1)* | 9.1 (5.5-14.7) | 68.6 (56.5-78.7)* |
| General Retailers | 0.0 | 69.4 (28.0-93.0) | 4.3 (2.1-8.6) | 15.9 (6.3-34.6)* |
| **TOTAL** | **24.8 (19.5-31.0)** | **71.6 (62.1-79.6)*** | **25.8 (23.6-28.0)** | **68.3 (57.8-77.1)*** |
|  | **Non-quality-assured ACTs** | | | |
| Public Health Facilities | 16.0 (1.0-77.6) | 14.5 (1.8-61.5) | 4.2 (1.4-12.2) | 5.1 (1.6-15.0) |
| Private Health Facilities | 57.8 (32.3-79.8) | 56.7 (32.8-77.9) | 25.4 (16.5-36.8) | 29.2 (7.8-66.2) |
| Specialised Drug Sellers | 35.9 (27.4-45.5) | 49.2 (33.6-65.0) | 9.7 (3.1-26.6) | 15.4 (6.5-32.1) |
| General Retailers | 0.0 | 31.3 (4.1-83.1) | 1.0 (0.2-3.4) | 0.0 |
| **TOTAL** | **36.6 (30.3-43.3)** | **48.9 (34.8-63.2)** | **7.3 (3.3-15.2)** | **11.7 (5.2-24.4)** |
|  | **Artemisinin monotherapies** | | | |
| Public Health Facilities | 16.0 (1.0-77.6) | 17.2 (2.2-66.2) | 0.0 | 0.0 |
| Private Health Facilities | 33.0 (9.3-70.2) | 20.7 (8.9-41.1) | 11.4 (3.2-33.3) | 28.1 (4.7-75.5) |
| Specialised Drug Sellers | 5.7 (4.1-7.8) | 4.1 (2.3-6.1) | 0.2 (0.0-0.7) | 1.1 (0.2-5.2) |
| General Retailers | 0.0 | 0.0 | 0.0 | 0.0 |
| **TOTAL** | **8.6 (4.2-16.9)** | **5.9 (3.8-8.9)** | **0.9 (0.3-2.6)** | **1.6 (0.4-6.4)** |
|  | **Non-artemisinin therapies** | | | |
| Public Health Facilities | 84.0 (68.0-92.9) | 100* | 97.9 (85.4-99.7) | 73.5 (56.3-85.6)* |
| Private Health Facilities | 92.5 (67.5-98.6) | 100 | 100 | 100 |
| Specialised Drug Sellers | 100 | 99.2 (95.7-99.9) | 99.4 (95.9-99.9) | 100 |
| General Retailers | 100 | 74.3 (21.1-96.9) | 97.0 (91.0-99.0) | 100 |
| **TOTAL** | **98.5 (95.7-99.5)** | **99.0 (96.6-99.7)** | **98.4 (95.6-99.4)** | **92.4 (85.3-96.3)*** |

Brackets denote 95% confidence intervals

*denotes p<0.05 for change over time

Source: Outlet surveys in 2010 and 2011
