# Supplementary material for: Has Tanzania Embraced the Green Leaf? Results from Outlet and Household Surveys before and after Implementation of the Affordable Medicines Facility -Malaria
Source: PLoS One. 2014 May 9;9(5):e95607. doi: 10.1371/journal.pone.0095607 (PMC4015933; doi:10.1371/journal.pone.0095607)
Supplement: Annex S2 — Median retail price per Adult Equivalent Treatment Dose of antimalarial drugs in tablet form in specialised drug sellers at baseline and endline by urban and rural areas (2010 USD). (DOCX) [file pone.0095607.s002.docx]

**Annex S2.** Median retail price per Adult Equivalent Treatment Dose of antimalarial drugs in tablet form in specialised drug sellers at baseline and endline by urban and rural areas(2010 USD)

|  | **Baseline** | | **Endline** | |
| --- | --- | --- | --- | --- |
|  | N | Median price [IQR] | N | Median price [IQR] |
|  | **Quality-assured ACTs** | | | |
| Urban | 256 | 7.04 [5.63 – 9.51] | 1,588 | 1.25 [0.75 – 2.50]* |
| Rural | 21 | 1.41 [0.85 – 3.52] | 207 | 0.94 [0.62 – 1.25] |
|  | **Non-quality-assured ACTs** | | | |
| Urban | 1,059 | 8.45 [6.69 – 13.52] | 1,689 | 7.50 [3.75 – 12.66] |
| Rural | 71 | 6.81 [3.52 – 9.16] | 92 | 6.25 [2.50 – 14.96] |
|  | **Non-artemisinin therapies** | | | |
| Urban | 1,504 | 1.06 [0.70 – 1.27] | 2,341 | 0.94 [0.70 – 1.41] |
| Rural | 456 | 0.85 [0.53 – 1.06] | 379 | 0.94 [0.56 – 0.94] |

IQR: Inter quartile range

*denotes p<0.05 for change over time

Artemisinin monotherapies not presented due to low numbers obtained

Source: Outlet surveys in 2010 and 2011
