# Supplementary material for: Has Tanzania Embraced the Green Leaf? Results from Outlet and Household Surveys before and after Implementation of the Affordable Medicines Facility -Malaria
Source: PLoS One. 2014 May 9;9(5):e95607. doi: 10.1371/journal.pone.0095607 (PMC4015933; doi:10.1371/journal.pone.0095607)
Supplement: Annex S3 — Market share by antimalarial category: percent distribution of antimalarial sales volumes by antimalarial category at baseline and endline by rural and urban areas. (DOCX) [file pone.0095607.s003.docx]

**Annex S3**: Market share by antimalarial category. Percent distribution of antimalarial sales volumes by antimalarial category at baseline and endline by rural and urban areas

|  | Quality-assured ACTs | Non-quality-assured ACTs | Artemisinin monotherpies | Non-artemisinin therapies |
| --- | --- | --- | --- | --- |
| Public HFs Urban Baseline | 89.8 | 7.0 | 1.7 | 1.4 |
| Public HFs Urban Endline | 75.8 | 0.3 | 0.2 | 23.8 |
| Public HFs Rural Baseline | 37.9 | 0.4 | 0.0 | 61.6 |
| Public HFs Rural Endline | 55.4 | 0.2 | 0.0 | 44.4 |
| Private HFs Urban Baseline | 23.0 | 14.4 | 0.5 | 62.1 |
| Private HFs Urban Endline | 36.5 | 13.1 | 0.5 | 49.9 |
| Private Hfs Rural Baseline | 13.4 | 6.4 | 0.0 | 80.2 |
| Private HFs Rural Endline | 13.7 | 0.6 | 0.0 | 85.6 |
| Specialised Drug Sellers Urban Baseline | 2.9 | 13.0 | 0.2 | 83.9 |
| Specialised Drug Sellers Urban Endline | 29.6 | 10.1 | 0.0 | 60.3 |
| Specialised Drug Sellers Rural Baseline | 1.0 | 1.5 | 0.0 | 97.5 |
| Specialised Drug Sellers Rural Endline | 38.6 | 2.7 | 0.0 | 58.7 |
| Urban Baseline | 32.2 | 11.3 | 0.6 | 55.8 |
| Urban Endline | 34 | 9.7 | 0 | 56.2 |
| Rural Baseline | 20.5 | 2.1 | 0.0 | 77.4 |
| Rural Endline | 45.9 | 1.1 | 0 | 52.9 |

HFs: Health facilities

General Retailers not presented separately due to low numbers obtained.

Source: Outlet surveys in 2010 and 2011
