# Supplementary material for: Has Tanzania Embraced the Green Leaf? Results from Outlet and Household Surveys before and after Implementation of the Affordable Medicines Facility -Malaria
Source: PLoS One. 2014 May 9;9(5):e95607. doi: 10.1371/journal.pone.0095607 (PMC4015933; doi:10.1371/journal.pone.0095607)
Supplement: Annex S4 — Description of household survey sample at baseline and endline by region and urban and rural areas. (DOCX) [file pone.0095607.s004.docx]

**Annex S4.** Description of household survey sample at baseline and endline by region and urban and rural areas

|  | **Baseline** | | | | | | | | | | | **Endline** | | | | | | | | | | | |
| --- | --- | --- | --- | --- | --- | --- | --- | --- | --- | --- | --- | --- | --- | --- | --- | --- | --- | --- | --- | --- | --- | --- | --- |
|  | Mwanza | Mbeya | Mtwara | | Urban | | | Rural | | Total | | Mwanza | | Mbeya | | | Mtwara | | Urban | Rural | | Total | |
| **Description of households:** | | | | | | | | | | | | | | | | | | | | | | | |
| HHs that participated | 1,945 | 1,640 | 1,945 | 763 | | | 4,660 | | | 5,423 | | 1,835 | 1,819 | | | 1,857 | | 842 | | | 4,669 | | 5511 |
| **Description of household members:** | | | | | | | | | | | | | | | | | | | | | | | |
| HH members interviewed | 9,702 | 5,547 | 5,625 | 2,643 | | | 18231 | | 20847 | | 8,834 | | 5,941 | | | 5,327 | | 2800 | | | 17302 | | 20102 |
| Percentage interviewed who were male | 43.6 | 41.6 | 43.0 | 37.7 | | | 40.7 | | 42.9 | | 44.0 | | 42.7 | | | 42.0 | | 44.1 | | | 43.7 | | 43.2 |
| HH members with study RDT results | 9,740 | 5,293 | 5,244 | 2,506 | | | 17771 | | 20277 | | 7,135 | | 4,493 | | | 4,220 | | 2339 | | | 15848 | | 18187 |
| Percentage parasite positive by study RDT | 23.7 | 2.4 | 23.0 | 3.7 | | | 20.7 | | 17.5 | | 16.1 | | 2.3 | | | 17.4 | | 3.5 | | | 13.5 | | 12.0 |
| **Socio-demographic characteristics of households:** | | | | | | | | | | | | | | | | | | | | | | | |
| **Occupation of household head:** | | | | | | | | | | | | | | | | | | | | | | | |
| Agriculture | 76.1 | 80.5 | 92.0 | 37.7 | | 91.5 | | | 82.1 | | 80.2 | | 81.7 | | | 88.2 | | 39.3 | | | 89.3 | | 82.6 |
| Unskilled manual labour | 7.0 | 3.9 | 1.6 | 14.6 | | 2.3 | | | 4.4 | | 3.9 | | 3.7 | | | 3.4 | | 13.4 | | | 2.2 | | 3.7 |
| Skilled manual labour | 3.8 | 3.3 | 1.2 | 10.6 | | 1.2 | | | 2.9 | | 2.9 | | 1.7 | | | 1.9 | | 5.9 | | | 1.6 | | 2.2 |
| Domestic service | 2.9 | 1.1 | 0.8 | 7.8 | | 0.4 | | | 1.7 | | 1.5 | | 1.5 | | | 0.8 | | 5.4 | | | 7.4 | | 1.4 |
| Sales and services | 7.1 | 6.7 | 2.5 | 21.7 | | 2.4 | | | 5.7 | | 6.2 | | 7.2 | | | 2.9 | | 21.5 | | | 3.5 | | 5.9 |
| Clerical | 0.3 | 0 | 0.3 | 0.5 | | 0.1 | | | 0.2 | | 0.3 | | 0 | | | 0 | | 0.3 | | | 0 | | 0 |
| Professional/  technical/  managerial | 2.9 | 4.2 | 1.6 | 7.3 | | 2.1 | | | 3.0 | | 5.1 | | 4.2 | | | 2.8 | | 14.4 | | | 2.6 | | 4.2 |
| **Education status of household head:** | | | | | | | | | | | | | | | | | | | | | | | |
| None | 23.1 | 26.3 | 29.4 | 12.1 | | 29.0 | | | | 26.0 | | 29.0 | 28.0 | | 28.8 | | | 15.5 | | | 30.6 | | 28.5 |
| Primary Incomplete | 15.1 | 14.1 | 18.9 | 10.4 | | 17.0 | | | | 15.8 | | 16.9 | 13.4 | | 16.4 | | | 12.9 | | | 15.8 | | 15.4 |
| Completed Primary | 54.3 | 52.3 | 48.2 | 59.2 | | 50.3 | | | | 51.9 | | 47.4 | 52.3 | | 50.2 | | | 54.5 | | | 49.3 | | 50.0 |
| Completed 4 years of secondary or higher | 7.5 | 7.3 | 3.5 | 18.3 | | 3.7 | | | | 6.3 | | 6.7 | 6.4 | | 4.6 | | | 17.1 | | | 4.3 | | 6.1 |

Source: Household surveys in 2010 and 2012
