# Supplementary material for: Has Tanzania Embraced the Green Leaf? Results from Outlet and Household Surveys before and after Implementation of the Affordable Medicines Facility -Malaria
Source: PLoS One. 2014 May 9;9(5):e95607. doi: 10.1371/journal.pone.0095607 (PMC4015933; doi:10.1371/journal.pone.0095607)
Supplement: Annex S5 — First treatment sought for febrile illness at baseline and endline, by region and urban and rural areas. (DOCX) [file pone.0095607.s005.docx]

**Annex S5**: First treatment sought for febrile illness at baseline and endline, by region and urban and rural areas

|  | Specialised drug seller | General retailer | Public health facility | Private health facility | Other ** |
| --- | --- | --- | --- | --- | --- |
| Mwanza Baseline | 53.3 | 13.6 | 18.7 | 5.8 | 8.6 |
| Mwanza Endline | 60.3 | 7.8 | 15.7 | 5.4 | 10.9 |
| Mbeya Baseline | 34.0 | 9.0 | 33.5 | 8.8 | 14.8 |
| Mbeya Endline | 48.6 | 9.1 | 19.3 | 8.1 | 15 |
| Mtwara Baseline | 13.7 | 34.8 | 35.4 | 7.4 | 8.8 |
| Mtwara Endline | 24.8 | 32.8 | 19.4 | 5.5 | 17.5 |
| Urban Baseline | 58.9 | 4.7 | 17.6 | 11.8 | 7.0 |
| Urban Endline | 62.8 | 2.0 | 14.5 | 12.8 | 8.0 |
| Rural Baseline | 36.9 | 19.5 | 27.2 | 5.5 | 10.9 |
| Rural Endline | 52.2 | 12.5 | 17.3 | 4.5 | 13.5 |

* denotes p<0.05 for change over time

**Other includes seeking treatment from home, a neighbor or a traditional healer

Source: Household surveys in 2010 and 2012
