# Supplementary material for: Has Tanzania Embraced the Green Leaf? Results from Outlet and Household Surveys before and after Implementation of the Affordable Medicines Facility -Malaria
Source: PLoS One. 2014 May 9;9(5):e95607. doi: 10.1371/journal.pone.0095607 (PMC4015933; doi:10.1371/journal.pone.0095607)
Supplement: Annex S6 — Treatment obtained for malaria at baseline and endline, by region and urban and rural areas. (DOCX) [file pone.0095607.s006.docx]

**Annex S6**: Treatment obtained for malaria at baseline and endline, by region and urban and rural areas

|  | **Baseline** | **Endline** |
| --- | --- | --- |
|  | **Any antimalarial** | |
| Mwanza | 42.6 (37.4-47.9) | 40.3 (36.0-44.8) |
| Mbeya | 34.7 (27.7-42.4) | 26.3 (21.5-31.8) |
| Mtwara | 42.3 (35.6-49.2) | 30.5 (24.5-37.2)* |
| Urban | 45.9 (37.3-54.8) | 47.9 (38.5-57.4) |
| Rural | 39.3 (35.3-43.5) | 33.6 (30.5-36.9)* |
|  | **ACT** | |
| Mwanza | 18.6 (14.7-23.2) | 21.3 (18.7-24.2) |
| Mbeya | 19.9 (15.2-25.6) | 18.1 (13.8-23.4) |
| Mtwara | 28.1 (22.3-34.8) | 22.6 (17.4-28.7) |
| Urban | 16.3 (11.2-23.0) | 22.7 (17.4-29.0) |
| Rural | 21.8 (18.6-25.5) | 20.3 (18.0-22.8) |
|  | **ACT out of those who obtained an antimalarial** | |
| Mwanza | 43.7 (36.3-51.4) | 52.9 (47.1-58.6) |
| Mbeya | 57.4 (45.5-68.4) | 68.9 (57.9-78.2) |
| Mtwara | 66.5 (55.8-75.8) | 74.1 (61.4-83.7) |
| Urban | 35.4 (26.3-45.6) | 47.4 (38.4-56.6) |
| Rural | 55.5 (49.3-61.7) | 60.5 (55.0-65.9) |

Brackets denote 95% confidence intervals

*denotes p<0.05 for change over time

Source: Outlet surveys in 2010 and 2012
